# Improving cardiorespiratory fitness and quality of life among heart failure patients: A comparative study of circuit resistance training and myofascial release techniques

**DOI:** 10.1371/journal.pone.0299348

**Published:** 2024-11-21

**Authors:** Sergio R. Thomaz, Cássia Da Luz Goulart, Natália Turri-Silva, Felipe A. Teixeira, Luis Freitas, Glauco Lima Rodrigues, Graziella França B. Cipriano, Gerson Cipriano, Lawrence P. Cahalin

**Affiliations:** 1 Physical Therapy Department, University of Brasilia, Brasilia, Federal District, Brazil; 2 Health Sciences and Technologies Graduate Program, University of Brasilia (UnB), Brasilia, Federal District, Brazil; 3 Escuela de Osteopatia de Madrid - Brasil e Internacional, Brasilia, Federal District, Brazil; 4 Graduate Program in Human Movement and Rehabilitation of Evangelical University of Goias, Goias, Brazil; 5 Department of Physical Therapy, Miller School of Medicine, University of Miami, Miami, Florida, United States of America; Kurume University School of Medicine, JAPAN

## Abstract

**Background:**

Heart failure (HF) imposes limitations due to severe dyspnea and fatigue, which are often linked to diminished exercise tolerance, potentially influenced by compromised microvascular density, blood flow, and muscle strength. Myofascial release techniques (MRT) have demonstrated the capacity to enhance blood flow by reducing fascial tension.

**Purpose:**

To assess the impact of incorporating MRT into Circuit Resistance Training (CRT) in comparison to an unsupervised home-based rehabilitation (RUHB) program on exercise tolerance (ET), muscle strength (MS), quality of life (QoL), and depression in patients with HF.

**Methods:**

A randomized clinical trial involved HF patients with reduced ejection fraction (HFrEF, ejection fraction <50%) and NYHA classes II–IV. Participants were randomly assigned to either CRT (performing 2 circuits of 8 exercises thrice a week for three months) or CRT+MRT (receiving a combination of CRT and 6 MRT interventions once a week). Assessments included cardiopulmonary exercise tests (CPET) to measure ET, MS evaluated through One Repetition Maximum (1RM), QoL using the Minnesota Living with HF Questionnaire (MLwHFQ), and Depression through the Beck Depression Inventory (BDI) conducted before and after the intervention**s.**

**Results:**

Thirty-eight patients (14 in CRT, 14 in CRT+MRT, and 10 in RUHB), with a mean age of 55 years and 50% male, completed the study. After 12 weeks, only the CRT group displayed a significant effect in certain ET variables VO_2_ peak [baseline 12 (9–15) vs post 16 (11–19) ml/kg/min, p<0.05], VO_2peak_ (ml/min) [baseline 848 (640–1056) vs post 1103 (852–1355) p<0.05], VE/VCO_2_ slope [baseline 34 (27–41) vs post 31 (27–36) p<0.05] and VO_2_/HR_peak_ [baseline 7 (5–9) vs post 11 (8–14) p<0.05]. There were significant decreases in the ΔMLwHFQ in the CRT group vs. RUHB (p<0.001) and CRT+MRT group vs. RUHB (p<0.001), demonstrating improved quality of life after 12 weeks in CRT and CRT+MRT groups.

**Conclusion:**

Our findings suggest that CRT alone is sufficient to enhance cardiorespiratory function and muscle capacity, improve the quality of life, and alleviate depression in individuals with HF.

## Introduction

Resistance training (RT) was described for the first time as a safe and vital exercise modality for heart failure patients according to American Heart Association recommendations only in the 2000s, with a favorable effect not only in muscular strength but also endurance, functional capacity, independence, and quality of life with positive effects in cardiovascular disease (CVD) individuals, mainly on those with frailty, a high prevalent condition in this patients, increasing their morbimortality rates [1–4] Accordingly, various resistance exercise modalities have been examined in this population, aiming to produce other possible benefits and extend exercise possibilities [5–7], crucial to maintaining exercise adherence, which is also a noteworthy problem in this population [5].

Previously denominated as circuit weight training, circuit resistance training (CRT) has emerged as an alternative for the elderly population, with positive effects on muscle strength, fat mass, lean body mass, and exercise capacity in both healthy adults and the elderly population [8, 9]. Several studies have examined the effects of the CRT modality in patients with heart failure (HF) and also found improvements in mitochondrial ATP production rate, which was correlated to increased peak oxygen consumption (VO_2peak_), skeletal muscle oxidative capacity, and lactate threshold [10–12].

Because the role of reduced peripheral blood flow appears to have a crucial influence on the pathophysiology of HF, adjuvant treatments aimed at improving blood flow have the potential to produce important clinical outcomes in HF patients. A particular manual therapy technique has been reported to improve vascular flow, such as myofascial release techniques (MRT) and balance diaphragmatic tensions technique (BDT). MRT can potentially improve autonomic nervous system balance and release the tension in the fascia of patients performing exercise [13–16].

This may be particularly important for HF patients performing CRT because arteries and veins pass through the fascia, which, if tight, has the potential to constrict vascular structures of muscles; therefore, an improvement in vascular flow may reduce muscle fatigue and result in better cardiorespiratory capacity. Also, the techniques focused on balancing diaphragmatic tensions may improve blood flow dynamics in HF patients during physical exercises since this patient presents an imbalanced intra-abdominal pressure gradient with altered tension in the diaphragms, which potentially decrease the fluids in the aortic hiatus, inguinal and adductor canals and to affect blood flow dynamics [14–16].

To the best of our knowledge, no previous studies have evaluated CRT alone and combined with MRT on several relevant clinical variables, including cardiorespiratory capacity, muscular performance, quality of life, and depression in HF patients following a cardiac rehabilitation program, especially as compared with Home-based program. In spite of evidence for the benefits of cardiac rehabilitation, some patients do not have access to supervised exercise in a cardiac rehabilitation program. Home-based programs, therefore, offer an opportunity to widen patient access and participation. There is evidence that home-based cardiac rehabilitation results in short-term improvements in exercise capacity and health-related quality of life of heart failure patients compared to usual care [13].

Thus, this study aimed to evaluate the effects of MRT and BDT combined with CRT compared with CRT alone on the cardiorespiratory capacity, muscular performance, depression, and quality of life of patients with HF compared with of the rehabilitation unsupervised home-base (RUHB).

## Materials and methods

### Subjects and design

The study was a single-blinded randomized clinical trial using a sample from the University of Brasília Cardiac Rehabilitation Program, who received CRT or CRT+MRT accordingly, as described below.

Our inclusion criteria were: 1) diagnosis of HF [17] of any etiology documented in the last six months; 2) left ventricular systolic dysfunction demonstrated by echocardiography (<50%); 3) *New York Heart Association classification* class II, III and IV) no previous participation in exercise three months before the study protocol. The individuals were with drug therapy optimized before the study. Our exclusion criteria were individuals previously diagnosed with moderate or severe chronic obstructive pulmonary disease, recent heart surgery in the last three months, morbid obesity (BMI ≥ 40 kg/m^2^), peripheral vascular disease, or inability to perform exercise.

### Ethical aspects

The study is registered on the Brazilian Clinical Trials Registry (number: RBR-4CMXRY) and was approved by the Ethics Committee and Research to Humans at the University of Brasilia (CAAE: 39564614.3.0000.0030). This adhered to the principles outlined in the Declaration of Helsinki and the Good Clinical Practice guidelines of the International Conference on Harmonization. Data collection took place between July 2015 and November 2016. Information about the objectives, procedures, and potential risks was provided to all participants, and a consent form was signed before the start of the proposed activities.

### Experimental procedure

Initially, all subjects underwent a clinical assessment by a medical cardiologist (A.L.), after which subjects provided informed consent. All the subjects underwent an incremental cardiorespiratory exercise test (CPX), after which patients were randomly assigned to either the CRT+MRT or CRT group. Randomization was done through the website https://www.random.org/. Seven days after the CPX, subjects underwent a skeletal muscle strength assessment via 1-RM and completed the Quality of Life assessment through the Minnesota Living with Heart Failure Questionnaire (MLWHFQ) and Beck depression inventory.

The subjects were randomly divided into either the CRT+MRT or CRT group. The subjects who declined, for any reason, to participate in the supervision program and those who have finished the CRT program and would like to participate in the home-based program were invited to enroll at least in the RUHB group, who were also monitored.

Every CRT session began and concluded with a 5 to 10-minute warm-up comprising stretching and global light exercises. Each CRT exercise was performed for 30 seconds and was followed by a 30-second rest period. Eight CRT exercises were performed in two separate circuits, with the first circuit consisting of knee extension and flexion, rowing, and elbow extension. The second circuit consisted of the calf muscle (plantar flexion), latissimus dorsi (lat-pull-down), pectoralis major (chest press), and abdominal muscle exercises. Each circuit consisted of 3 sets of 8 to 12 repetitions of each exercise at 60–80% of 1-RM load with the heart rate between the anaerobic threshold and respiratory compensation point obtained from the CPX. A one-minute rest period was given between the first and second circuits. Initially, CRT intensity was set at 60% of 1-RM, which was increased to 70% and 80% of 1-RM every four weeks. The number of repetitions was increased from 8 to 12 every week. Patients were asked not to change their normal activities of daily living during the 12-week period [18, 19].

The HR was monitored continuously (Polar H7 sensor and Polar Beat app, Polar Electro Inc., Kempele, Finland) and (IPAD Air 2, Apple, Cupertino, USA) during CRT and the recovery period.^32^ The systolic blood pressure (SBP) and diastolic (DBP) were measured using an automated oscillometric calibrated device (OMROM MIT Elite plus, OMROM Health Care Inc, IL, USA) before and after each CRT session.

#### Description of the techniques of MRT

The MRT was based on the treatment described in the study of Jardine WM et al. [14]. The evaluations and treatments were performed by an osteopath with five years of experience. The evaluation was performed initially with tests and palpations in specific areas of the body to evaluate the mobility and collect basic information on tissue characteristics (also known as TART–for Tenderness, Asymmetry, Range of motion change, Tissue texture change) to identify patterns of stiffness/tenderness of the tissues (muscles, fascias, etc). The evaluation also included tests such as sitting diaphragm evaluation, sphenobasilar symphysis (SBS) ’listening’, sacral ’listening’, pelvic floor evaluation, and global femoral artery evaluation. The evaluation was performed bilaterally, but treatment techniques were performed only on the side where restriction was found.

The MRT consisted of six selected osteopathy techniques (cranial, myofascial, and visceral techniques) are described in Table 1. Each technique was performed for 2 minutes with patients mostly supine, with an entire completed session lasting 15 minutes, 1x/week for 12 weeks.

**Table 1:** Description of the myofascial release techniques.

**1.Thoracic diaphragm release technique**	Bilateral hand contact in the subcostal region was applied to move the thorax into flexion/extension and lateral rotation to identify the area of greatest resistance. The direction identified to have the greatest resistance underwent a stretch and held until release of the tension after which the thorax was returned to the initial position with the patient sitting.
**2.Tentorium cerebelli release technique**	A reciprocal tension maintaining a bilateral external rotation of the temporal bones was performed with the dura mater moved in cephalic traction and held until release with the patient in the supine position with a pillow under the knees.
**3. Pelvic floor release technique**	Palpation of the sacrum and low back was performed while focusing on breathing. During each expiration one sacrum traction was performed to the side with the greatest resistance and repeated until fascial release. The patient was supine with knees flexed.
**4.Iliac fascial release technique**	The release of the iliac fascia was performed with the fingers of the therapist’s hands pressing on the anterior superior iliac spine in a diagonal, inferior, and medial direction while the patient was supine and knees in flexion. The pressure was held until release.
**5. Femoral artery release technique**	After the therapist localized the femoral artery both hands were placed around it and provided both a cranial and caudal pressure after which the direction identified to have the greatest resistance underwent a stretch and was held until release. The patient was supine with a pillow under the knees.
**6.Balancing the three diaphragms technique**	One hand in the thoracic region (subcostal) and the other on the temporal bone allowed each breath to be palpated after which synchronization of diaphragms was performed using gentle movement of both hands to facilitate optimal breathing. The procedure was repeated with one hand in the chest (mid-sternal) and the other near the pelvic floor on the sacrum. This was repeated in each position until synchronization of the subcostal, mid-sternal, and pelvic floor was achieved. The patient was in the supine position with a pillow under the knees

Subjects in RUHB participated in the unsupervised home-based rehabilitation program that consisted of five reunions in three months. The subjects received guides about aerobic and resistance exercises to be performed at home, orientation about core components topics, including 1) exercise and heart rate monitoring, 2) nutrition, 3) pharmacology, 4) biopsychosocial behavior, and 5) risk factors. The patients were asked about their home exercise at each reunion, and any doubt was remedied. After 12 weeks of the CRT program and the home-based program, all subjects were submitted to the same test.

### Measurements and outcome variables

#### Cardiorespiratory exercise testing

A symptom-limited incremental ramp protocol of 10–15 watts/minute was used while respiratory gases were continuously obtained from a metabolic cart (Quark CPET, Cosmed, Rome, Italy), which was connected to an electromagnetic bike (Corival, Lode, Groningen, Netherlands). Before the CPX, the oxygen (O_2_), carbon dioxide (CO_2_), and flow sensors were calibrated. All CPX were supervised by a cardiologist (A.L.).

Before each test, for 5 minutes, they were allowed patients to adapt to the cycle ergometer and gas analyses. A 12-lead electrocardiogram was continuously monitored (Quark C12X, Cosmed, Rome, Italy) and recorded at the end of each minute. Blood pressure was obtained with a standard sphygmomanometer every 2 minutes during the test and up to 15 minutes after the CPX. Exhaled gases were collected breath by breath, providing oxygen consumption ($\dot{\text{V}}$O_2_)_,_ carbon dioxide production ($\dot{\text{V}}$CO_2_)_,_ minute ventilation ($\dot{\text{V}}$E), $\dot{\text{V}}$E/$\dot{\text{V}}$CO_2_ slope, and O_2_ pulse (VO_2_/HR_peak_) was calculated using the product of peak $\dot{V}$O_2_ and peak HR. Data collection continued for at least 15 minutes of passive recovery. The CPX was performed using stress test guidelines of the Brazilian Society of Cardiology [20].

#### Muscular strength test

1-RM of each of the 8 CRT exercises was performed following the procedure and the recommendations of Dias et al. [18]. The 1-RM was performed after the subject was familiarized with the CRT unit (two sessions up to 72 hours before the test) (ENRAF-NONIUS, the EN-DYNAMIC line, Netherlands).

Prior to testing, a warm-up on each exercise was performed with a load of approximately 50% of the 1-RM familiarization load. After two minutes of rest, there were three attempts with progressive loads, with rest intervals of three to five minutes. Subjects were asked to complete two repetitions; however, the load recorded as 1-RM was the greatest the individual could complete during a single repetition. The 1-RM of each exercise was used to provide the initial load (60% 1-RM). The 1-RM of each of the 8 CRT exercises was recorded individually and summed as a total composite score.

#### Minnesota living with heart failure questionnaire and Beck Depression Inventory

All patients were administered the Minnesota Living with Heart Failure Questionnaire (MLwHFQ) [21] and Beck Depression Inventory (BDI) [22] using standardized methods before and after 12 weeks.

#### Statistical analysis

Prior to statistical analysis, all variables will be tested for normality using the Shapiro-Wilk tests. Based on the nature of the distribution, the variables were analyzed parametrically or non-parametrically.

Comparative analyses included paired t-test or Friedman’s test with Dunn post hoc or one-way ANOVA with post hoc Bonferroni based on the data distribution. Two-way analysis of variance (ANOVA) for multiple comparisons was performed to compare baseline and post-rehabilitation in CRT, CRT+MRT, and UHBR. Statistical analyses were performed using GraphPad Prism (version 7.02) programs. The level of statistical significance was p <0.05.

## Results

**Fig 1:**
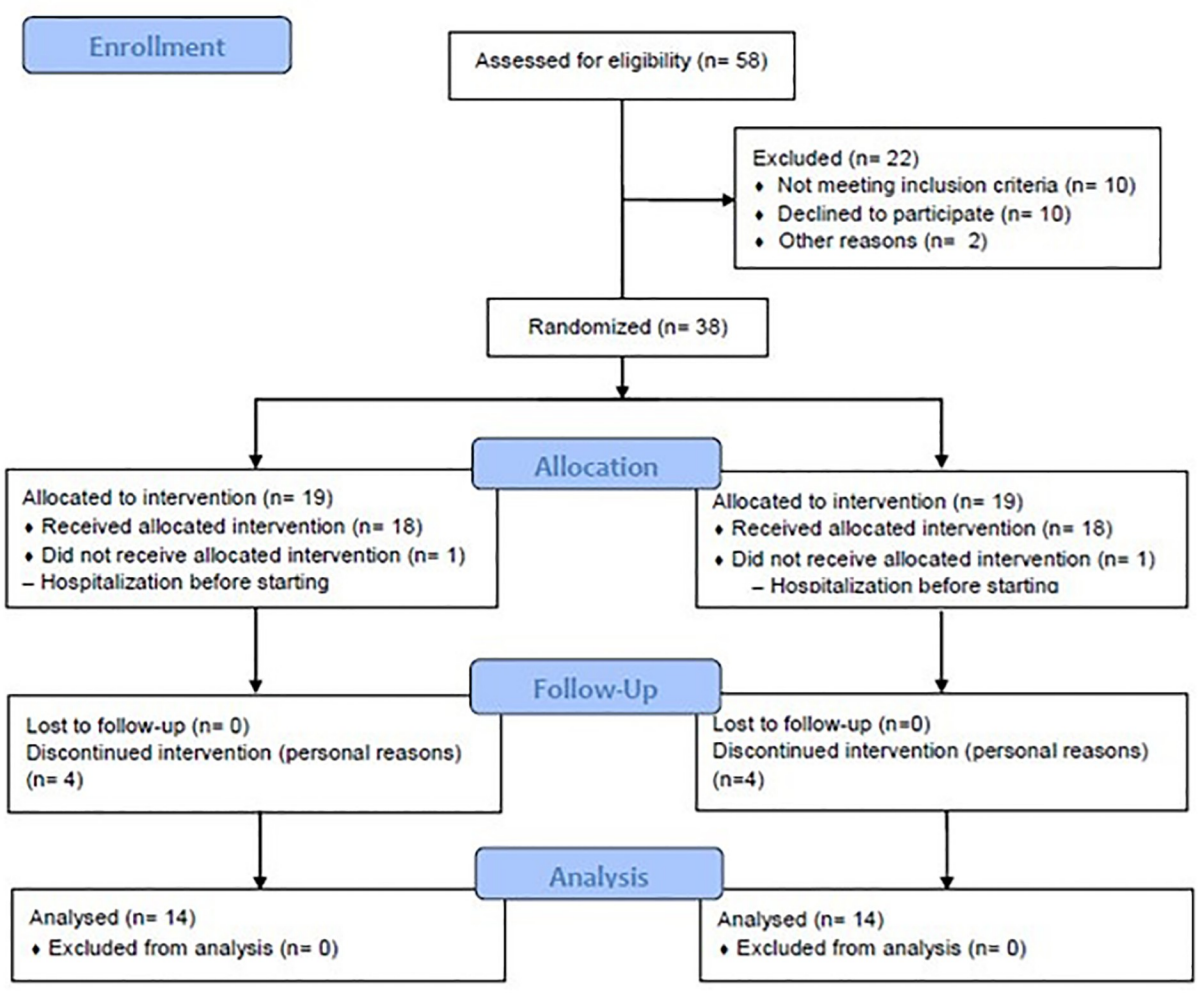
Study flow diagram.

Fifty-eight patients between the ages of 35 and 75 were included in the study. They had been diagnosed with heart failure of either ischemic or idiopathic origin, and their drug therapy had been optimized prior to the study. Twenty-two patients were excluded, and thirty-eight patients (14 subjects in each CRT group) completed 18 to 32 of 32 total CRT sessions in the 12-week study with an adherence rate of 77% for both groups. Ten patients in the RUHB completed the home-based program. No complication or adverse event occurred during or after CRT, CRT+MRT, or RUHB (Fig 1).

All subjects continued their antihypertensive and medical treatments throughout the study. They were all prescribed ACE inhibitors and beta-blockers. In addition, 14% and 50% of the CRT+MRT group were prescribed Digoxin and Furosemide, respectively. For the CRT group, 21% and 78% were prescribed Digoxin and Furosemide; for the RUHB group, 40% and 80% were prescribed Digoxin and Furosemide, respectively. Furthermore, statins were prescribed to 64% of the CRT+MRT group, 78% of the CRT group, and 40% of the RUHB group.

No significant differences in patient characteristics were observed between groups at baseline (Table 2). A summary of the cardiorespiratory dependent measures is shown in Table 3. After 12 weeks, the participants in the CRT groups improved the VO_2_ peak (ml/kg/min and ml/min), O_2_ pulse (VO_2_/HR_peak_), and VE/VCO_2_ slope (p<0.05) in the CRT+MRT and UHBR group we did not find a significant difference in cardiorespiratory parameters.

**Table 2:** Clinical characteristics of the study population.

Variables	CRT+MRT (n = 14)	CRT (n = 14)	RUHB (n = 10)	P value
Age (years)	52 (47–57)	60 (53–66)	58 (50–66)	0.134
Sex, male (%)	9 (64)	7 (50)	6 (60)	0.720
Weight (Kg)	74 (66–83)	76 (66–85)	67 (58–75)	0.311
Body Mass Index (kg/m^2^)	26 (23–29)	28 (25–30)	24 (22–26)	0.173
Ejection fraction (%)	37 (31–43)	34 (25–43)	35 (23–47)	0.823

Notes: Data are presented as mean and confidence interval. CRT–Circuit Resistance Training, MRT–Myofascial Release Techniques. P value = Anova one way.

**Table 3:** Cardiorespiratory variable measurement of the study population.

Variables	CRT+MRT (n = 14)		CRT (n = 14)		RUHB (n = 10)	
	Baseline	Post	Baseline	Post	Baseline	Post
VO_2peak (ml/min)_	1143 (919–1366)	1237 (1015–1458)	848 (640–1056)	1103 (852–1355)#	940 (755–1123)	909 (719–1099)
VO_2peak_ (ml/kg/min)	15 (13–17)	17 (14–19)	12 (9–15)	16 (11–19)#	15 (12–17)	15 (12–17)
VO_2_/HR_peak_	9 (7–11)	10 (8–12)	7 (5–9)	11 (8–14)#	9 (7–10)	9 (7–10)
VE/VCO_2_ slope	29 (26–33)	31 (26–35)	34 (27–41)	31 (27–36)#	31 (28–35)	31 (28–34)

Notes: Data are presented as mean and confidence interval. CRT–Circuit Resistance Training, MRT–Myofascial Release Techniques. P value = Anova two way.

*post CRT+MRT *vs* post CRT p<0.05

^†^post CRT+MRT *vs* post control p<0.05

^#^baseline vs post

**Fig 2:**
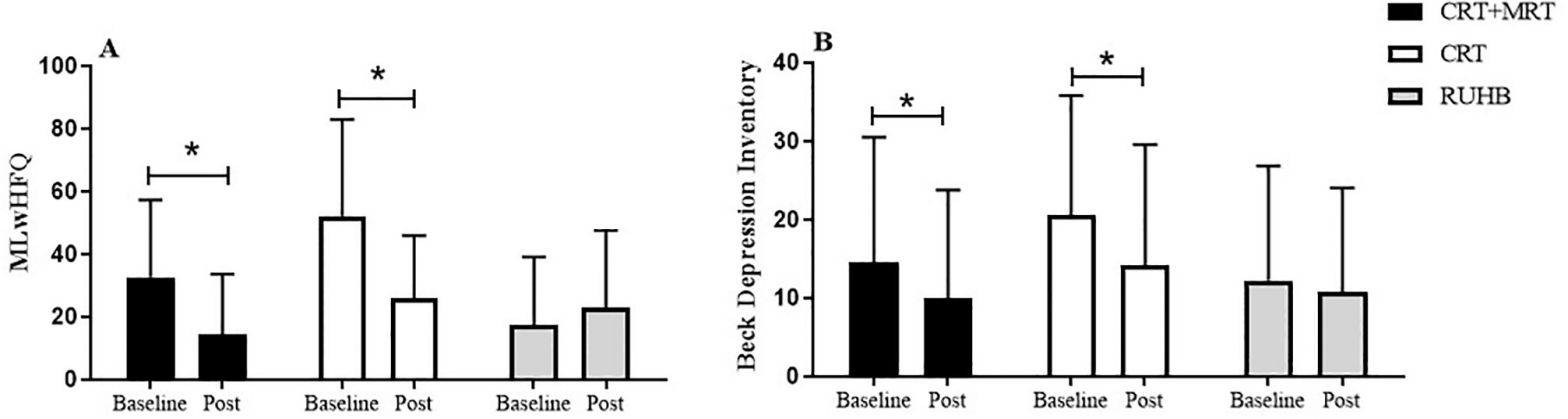
Quality of life and Beck Depression Inventory baseline and post CRT+MRT, CRT and RUHB. Data are presented as mean and standard deviation.

**Fig 3:**
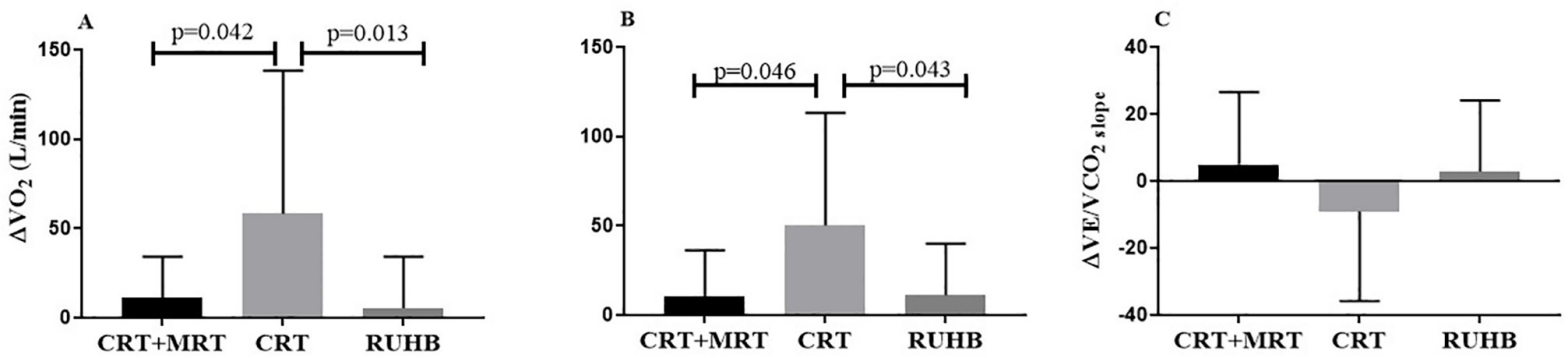
ΔVO_2_ (ml/min), ΔVO_2_/HR and ΔVE/VCO_2_ slope baseline and post CRT+MRT, CRT and RUHB. Data are presented as mean and standard deviation. Δ = baseline-post. *P <0.05.

In Fig 3, we observed that patients in the CRT and CRT+MRT group, after 12 weeks of intervention, showed an improvement in quality of life using the MLwHFQ (Fig 2A, p<0.05) and in depression rates using the BDI (Fig 2B, p <0.05).

There were significant increases in muscle strength (sum of all eight exercises) post CRT group (total weight, baseline 285.7±12.1 kg vs. post 336.5±14.9 kg, p<0.001) and CRT+MRT group (total weight, Kg, baseline 365±14.9 kg vs post 424.4±16.8 kg, p<0.001) (Table 4).

**Table 4:** Changes in maximal strength within each group after treatment.

Resistance exercise	CRT+MRT (n = 14)		CRT (n = 14)		RUHB (n = 10)	
	Baseline	Post	Baseline	Post	Baseline	Post
**Knee extension**	38.4±13.7	47.8±16.4†	26.1±9.6	31.9±12.2†	38.9±16.4	41.2±16.2
**Knee flexion**	38.9±11.0	45.4±12.9†	29.3±11.3	34.3±13.5†	38.1±14.2	38.5±13.3
**Rowing**	33.6±9.1	40.9±11.9†	27.4±9.5	32.3±13.1†	36.3±13.8	36.8±13.6
**Elbow extension**	50.1±12.8	52.5±10.2†	39.8±13.4	45.5±12.1†	50.7±11.7	50.2±10.0
**Chest press**	53.3±20.5	60.4±21.7†	39.3±18.1	46.8±22.8†	57.1±24.4	56.6±26.3
**Abdominal muscle**	37.6±11.2	45.5±14.0†	30.5±10.6	36.0±12.7†	39.5±13.5	38.6±13.7
**Calf muscle**	78.2±24.6	91.5±27.6†	62.8±21.7	76.1±21.1†	85.7±25.8	88.6±27.7
**Pull-down**	34.8±9.9	40.5±11.9†	30.6±9.8	33.7±10.1†	38.9±11.4	40.1±15.1
**Total weight, Kg**	365±14.9	424.4±16.8†	285.7±12.1	336.5±14.9†	385.2±16.8	390.8±17.5

Data are presented as mean and standard deviation. CRT–Circuit Resistance Training, MRT–Myofascial Release Techniques. Weight in Kilogram.

p value = test T pared (post/pre)

^†^ baseline vs post p<0.001

ANOVA of one-way shows a significant difference in CRT group vs. CRT+MRT group and CRT group vs. RUHB in both parameters ΔVO_2_ (Fig 3A) and ΔO_2_ pulse (ΔVO_2_/HR_peak_) (Fig 3B) (p<0.05) (Fig 3), in Fig 3C there was no significant difference in the ΔVE/VCO_2_ slope (p>0.05).

**Fig 4:**
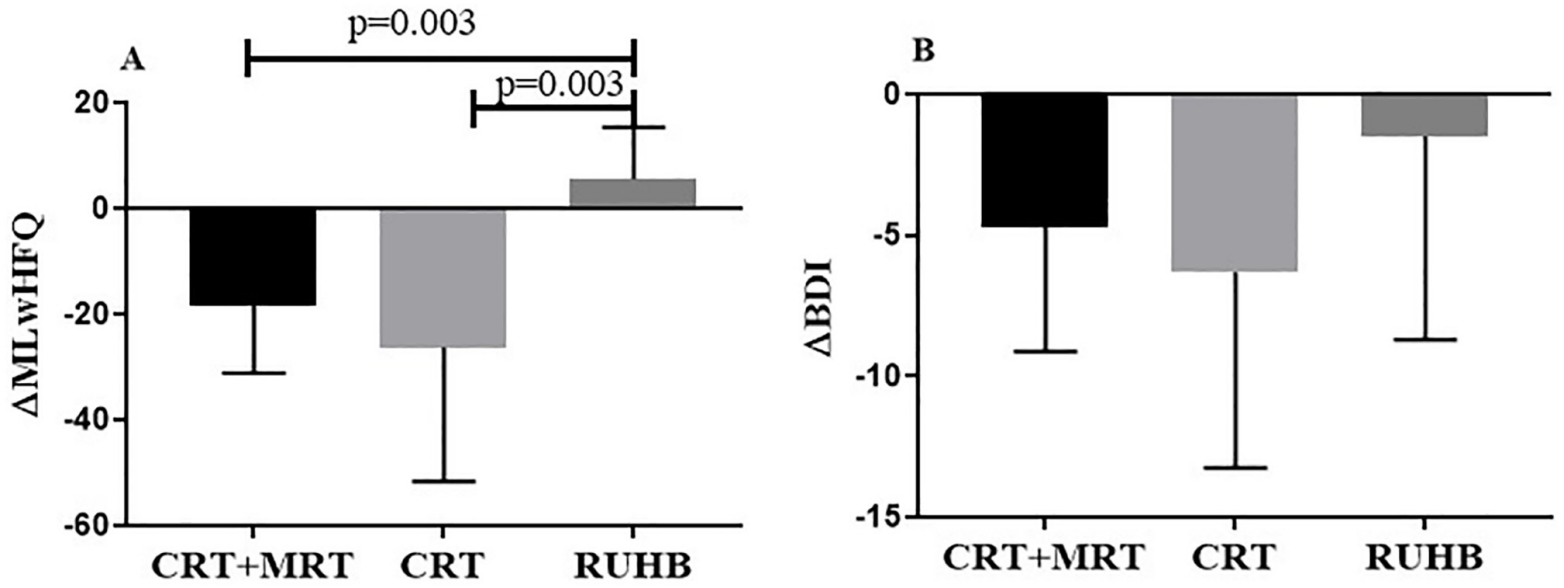
ΔQuality of life and ΔBeck Depression Inventory baseline and post CRT+MRT, CRT and RUHB. **Δ = pre-post**. *P <0.05.

There were significant decreases in the ΔMLwHFQ in the CRT group vs. RUHB (p<0.001) and CRT+MRT group vs. RUHB (p<0.001) (Fig 4), demonstrating improved quality of life after 12 weeks in CRT and CRT+MRT groups.

## Discussion

The primary objectives of this study were to assess the impact of CRT alone and CRT combined with MRT, in comparison to RUHB, on cardiorespiratory function, muscular performance, depression, and quality of life among patients with HF. Our findings revealed that the CRT group exhibited a safe and significant enhancement in VO_2peak_, O_2_pulse, muscle strength, and quality of life. Similarly, the CRT+MRT group demonstrated improvements in quality of life, BDI scores, and muscular performance capacity.

By utilizing circuit resistance training, keeping the heart rate levels between the anaerobic threshold and the respiratory compensation point—as determined by cardiopulmonary exercise testing (CPX)—the intervention successfully enhanced both muscle strength and cardiorespiratory fitness in heart failure (HF) patients. These improvements were concurrent and led to a significant enhancement in the patient’s quality of life, consistent with findings reported in systematic reviews within this field [23].

Our study protocol followed a recent CRT review [25] in that we used eight different CRT exercises, of which three sets were performed with a work-to-rest ratio of 30:30 seconds, 3x/week, at an initial resistance of 60% 1RM, which progressed to 80% of 1-RM. Several previous studies performed CRT, which often combined resistance training with aerobic exercise and produced similar results [17, 23, 24]. However, the resistance training used in these studies reflected substantial heterogeneity in the strength exercise protocols, the number of resistance exercises performed, the type and duration of aerobic exercise, and recovery intervals [17, 23, 24]. Our method of CRT was based on CRT review [25] and followed the recommendations of the American Heart Association, which is an essential distinction of our study and results.

A previous study of CRT in patients with HF was found that used the same CRT program as ours and resulted in significant increases in aerobic endurance and skeletal muscle strength [26]. Our findings underscore the positive role of resistance training, specifically CRT, in improving cardiorespiratory and skeletal muscle performance as well as quality of life in HF patients.

Factors leading to disability, such as a decline in muscular strength and VO_2peak_, are associated with a diminished quality of life in individuals with heart failure [27, 28]. In fact, Levinger et al. (2004) [29] found significant correlations between quality of life and post-training peak VO_2_ as well as total weight lifted during the post-maximal strength test in HF patients after a resistance training program [29]. In our study, like in the Levinger study [29], the MLwHFQ was used to measure quality of life, which, as a disease-specific instrument, appears to better capture change in quality of life in HF. Combined aerobic and resistance training was recently found to significantly improve the MLwHFQ scores in patients with HF.

To our knowledge, this is the first study that has investigated the effect of CRT on depression in HF patients. In a recent review, Rutledge et al. (2006) [1] were unable to develop definitive conclusions regarding the intervention effectiveness of depression in HF due to heterogeneous and small sample-sized studies.

Our results support the role of resistance training in HF and, in particular, how CRT safely improved cardiorespiratory and skeletal muscle performance and quality of life in patients with HF.

In this study, patients in the CRT+MRT group had improvements in quality of life, BDI, and muscular capacity performance post-rehabilitation; between the groups, we did not find additional improvements for CRT+MRT, demonstrating that CRT alone is sufficient to obtain cardiorespiratory improvement, muscular capacity performance, depression, and quality of life of patients with HF. These results suggest that MRT does not influence cardiorespiratory performance in HF patients. The most likely reasons for this finding include the sub-optimal provision of MRT, alterations in the autonomic nervous system and vascular regulation of HF patients, or HF medications. Nonetheless, no negative effects of MRT were reported or observed in this first study of MRT in HF patients.

### Limitations

Several minor limitations should be acknowledged, including the potential masking effects of HF medications on MRT outcomes, variations in the baseline condition of subjects despite randomization in the CRT alone group, and the non-randomization of the RUHB group. Future studies should explore the influence of HF medications on MRT and consider refining subject randomization procedures.

## Conclusions

This is the first study to examine the effects of MRT and CRT in patients with HF. The results affirm that CRT alone is sufficient to enhance cardiorespiratory function and muscular capacity, alleviate depression, and improve quality of life in HF patients.

## Supporting information

S1 Checklist CONSORT 2010 checklist of information to include when reporting a randomised trial*. (DOC)

S1 Protocol (DOCX)

## References

[1] Rutledge T, Reis VA, Linke SE, Greenberg BH, Mills PJ. Depression in Heart Failure. J Am Coll Cardiol. 2006;48: 1527–1537. doi: 10.1016/j.jacc.2006.06.055 17045884

[2] Jiang W, Alexander J, Christopher E, Kuchibhatla M, Gaulden LH, Cuffe MS, et al. Relationship of Depression to Increased Risk of Mortality and Rehospitalization in Patients With Congestive Heart Failure. Arch Intern Med. 2001;161: 1849. doi: 10.1001/archinte.161.15.1849 11493126

[3] Daullxhiu I, Haliti E, Poniku A, Ahmeti A, Hyseni V, Olloni R, et al. Predictors of exercise capacity in patients with chronic heart failure. Journal of Cardiovascular Medicine. 2011;12: 223–225. doi: 10.2459/JCM.0b013e328343e950 21285738

[4] Vogiatzis I, Zakynthinos S. The physiological basis of rehabilitation in chronic heart and lung disease. J Appl Physiol. 2013;115: 16–21. doi: 10.1152/japplphysiol.00195.2013 23620491

[5] Meka N, Katragadda S, Cherian B, Arora RR. Review: Endurance exercise and resistance training in cardiovascular disease. Ther Adv Cardiovasc Dis. 2008;2: 115–121. doi: 10.1177/1753944708089701 19124415

[6] Pearson MJ, Smart NA. Effect of exercise training on endothelial function in heart failure patients: A systematic review meta-analysis. Int J Cardiol. 2017;231: 234–243. doi: 10.1016/j.ijcard.2016.12.145 28089145

[7] Maiorana AJ, Naylor LH, Exterkate A, Swart A, Thijssen DHJ, Lam K, et al. The Impact of Exercise Training on Conduit Artery Wall Thickness and Remodeling in Chronic Heart Failure Patients. Hypertension. 2011;57: 56–62. doi: 10.1161/HYPERTENSIONAHA.110.163022 21059991

[8] O’Connor CM, Whellan DJ, Lee KL, Keteyian SJ, Cooper LS, Ellis SJ, et al. Efficacy and Safety of Exercise Training in Patients With Chronic Heart Failure. JAMA. 2009;301: 1439. doi: 10.1001/jama.2009.454 19351941 PMC2916661

[9] SAVAGE PA, SHAW AO, MILLER MS, VANBUREN P, LEWINTER MM, ADES PA, et al. Effect of Resistance Training on Physical Disability in Chronic Heart Failure. Med Sci Sports Exerc. 2011;43: 1379–1386. doi: 10.1249/MSS.0b013e31820eeea1 21233772 PMC3410739

[10] SELIG S, CAREY M, MENZIES D, PATTERSON J, GEERLING R, WILLIAMS A, et al. Moderate-intensity resistance exercise training in patients with chronic heart failure improves strength, endurance, heart rate variability, and forearm blood flow*1. J Card Fail. 2004;10: 21–30. doi: 10.1016/S1071-9164(03)00583-9 14966771

[11] Williams AD, Carey MF, Selig S, Hayes A, Krum H, Patterson J, et al. Circuit Resistance Training in Chronic Heart Failure Improves Skeletal Muscle Mitochondrial ATP Production Rate—A Randomized Controlled Trial. J Card Fail. 2007;13: 79–85. doi: 10.1016/j.cardfail.2006.10.017 17395046

[12] Degache F, Garet M, Calmels P, Costes F, Bathélémy J, Roche F. Enhancement of isokinetic muscle strength with a combined training programme in chronic heart failure. Clin Physiol Funct Imaging. 2007;27: 225–230. doi: 10.1111/j.1475-097X.2007.00741.x 17564671

[13] Zwisler A-D, Norton RJ, Dean SG, Dalal H, Tang LH, Wingham J, et al. Home-based cardiac rehabilitation for people with heart failure: A systematic review and meta-analysis. Int J Cardiol. 2016;221: 963–969. doi: 10.1016/j.ijcard.2016.06.207 27441476

[14] Jardine WM, Gillis C, Rutherford D. The effect of osteopathic manual therapy on the vascular supply to the lower extremity in individuals with knee osteoarthritis: A randomized trial. International Journal of Osteopathic Medicine. 2012;15: 125–133. doi: 10.1016/j.ijosm.2012.07.001

[15] Queré N, Noël E, Lieutaud A, d’Alessio P. Fasciatherapy combined with pulsology touch induces changes in blood turbulence potentially beneficial for vascular endothelium. J Bodyw Mov Ther. 2009;13: 239–245. doi: 10.1016/j.jbmt.2008.06.012 19524848

[16] Lombardini R, Marchesi S, Collebrusco L, Vaudo G, Pasqualini L, Ciuffetti G, et al. The use of osteopathic manipulative treatment as adjuvant therapy in patients with peripheral arterial disease. Man Ther. 2009;14: 439–443. doi: 10.1016/j.math.2008.08.002 18824395

[17] Mant J. Management of Chronic Heart Failure in Adults: Synopsis of the National Institute for Health and Clinical Excellence Guideline. Ann Intern Med. 2011;155: 252. doi: 10.7326/0003-4819-155-4-201108160-00009 21844551

[18] Dias RMR, Avelar A, Menêses AL, Salvador EP, da Silva DRP, Cyrino ES. Segurança, reprodutibilidade, fatores intervenientes e aplicabilidade de testes de 1-RM. Motriz: Revista de Educação Física. 2013;19: 231–242. doi: 10.1590/S1980-65742013000100024

[19] Seo D-I, Kim E, Fahs CA, Rossow L, Young K, Ferguson SL, et al. Reliability of the one-repetition maximum test based on muscle group and gender. J Sports Sci Med. 2012;11: 221–5. 24149193 PMC3737872

[20] Ghorayeb N, Stein R, Daher DJ, da Silveira AD, Ritt LEF, dos Santos DFP, et al. The Brazilian Society of Cardiology and Brazilian Society of Exercise and Sports Medicine Updated Guidelines for Sports and Exercise Cardiology—2019. Arq Bras Cardiol. 2019. doi: 10.5935/abc.20190048 30916199 PMC6424031

[21] Carvalho VO, Guimarães GV, Carrara D, Bacal F, Bocchi EA. Validação da versão em português do Minnesota Living with Heart Failure Questionnaire. Arq Bras Cardiol. 2009;93: 39–44. doi: 10.1590/S0066-782X2009000700008 19838469

[22] Gomes-Oliveira MH, Gorenstein C, Neto FL, Andrade LH, Wang YP. Validation of the Brazilian Portuguese Version of the Beck Depression Inventory-II in a community sample. Revista Brasileira de Psiquiatria. 2012;34: 389–394. doi: 10.1016/j.rbp.2012.03.005 23429809

[23] Pollock ML, Franklin BA, Balady GJ, Chaitman BL, Fleg JL, Fletcher B, et al. Resistance Exercise in Individuals With and Without Cardiovascular Disease. Circulation. 2000;101: 828–833. 10683360 10.1161/01.cir.101.7.828

[24] Zheng L, Pan D, Gu Y, Wang R, Wu Y, Xue M. Effects of high-intensity and moderate-intensity exercise training on cardiopulmonary function in patients with coronary artery disease: A meta-analysis. Front Cardiovasc Med. 2022;9. doi: 10.3389/fcvm.2022.961414 36204588 PMC9530785

[25] Romero-Arenas S. Impact of Resistance Circuit Training on Neuromuscular, Cardiorespiratory and Body Composition Adaptations in the Elderly. Aging Dis. 2013;04: 256–263. doi: 10.14336/AD.2013.0400256 24124631 PMC3794722

[26] Kelemen MH, Stewart KJ, Gillilan RE, Ewart CK, Valenti SA, Manley JD, et al. Circuit weight training in cardiac patients. J Am Coll Cardiol. 1986;7: 38–42. doi: 10.1016/s0735-1097(86)80256-x 3941214

[27] Belardinelli R, Georgiou D, Cianci G, Purcaro A. Randomized, Controlled Trial of Long-Term Moderate Exercise Training in Chronic Heart Failure. Circulation. 1999;99: 1173–1182. 10069785 10.1161/01.cir.99.9.1173

[28] DELAGARDELLE C, FEIEREISEN P, AUTIER P, SHITA R, KRECKE R, BEISSEL J. Strength/endurance training versus endurance training in congestive heart failure. Med Sci Sports Exerc. 2002;34: 1868–1872. doi: 10.1097/00005768-200212000-00002 12471289

[29] Levinger I, Bronks R, Cody D V., Linton I, Davie A. Resistance training for chronic heart failure patients on beta blocker medications. Int J Cardiol. 2005;102: 493–499. doi: 10.1016/j.ijcard.2004.05.061 16004896

