# Supplementary material for: Improving cardiorespiratory fitness and quality of life among heart failure patients: A comparative study of circuit resistance training and myofascial release techniques
Source: PLoS One. 2024 Nov 21;19(11):e0299348. doi: 10.1371/journal.pone.0299348 (PMC11581296; doi:10.1371/journal.pone.0299348)
Supplement: S1 Protocol — (DOCX) [file pone.0299348.s002.docx]

Initially, all volunteers underwent a clinical assessment by a medical cardiologist (A.L.), after which subjects provided informed consent. All the subjects underwent an incremental cardiorespiratory exercise test (CPX), after which patients were randomly assigned to either the CRT+MRT or CRT group. Randomization was done through the website <https://www.random.org/>. Seven days after the CPX, subjects underwent a skeletal muscle strength assessment via 1-RM and completed the Quality of Life assessment through the Minnesota Living with Heart Failure Questionnaire (MLWHFQ) and [Beck depression inventory](http://www.nctsnet.org/content/beck-depression-inventory-second-edition).

The subjects were randomly divided into either the CRT+MRT or CRT group. The volunteers who declined, for any reason, to participate in the supervision program and those who have finished the CRT program and would like to participate in the home-based program were invited to enroll at least in the URBH group, who were also monitored.

Every CRT session began and concluded with a 5 to 10-minute warm-up comprising stretching and global light exercises. Each CRT exercise was performed for 30 seconds and was followed by a 30-second rest period. Eight CRT exercises were performed in two separate circuits, with the first circuit consisting of knee extension and flexion, rowing, and elbow extension (Figure 1). The second circuit consisted of the calf muscle (plantar flexion), latissimus dorsi (lat-pull-down), pectoralis major (chest press), and abdominal muscle exercises. Each circuit consisted of 3 sets of 8 to 12 repetitions of each exercise at 60-80% of 1-RM load with the heart rate between the anaerobic threshold and respiratory compensation point obtained from the CPX. A one-minute rest period was given between the first and second circuits. Initially, CRT intensity was set at 60% of 1-RM, which was increased to 70% and 80% of 1-RM every four weeks. The number of repetitions was increased from 8 to 12 every week. Patients were asked not to change their normal activities of daily living during the 12-week period [18,19].

^
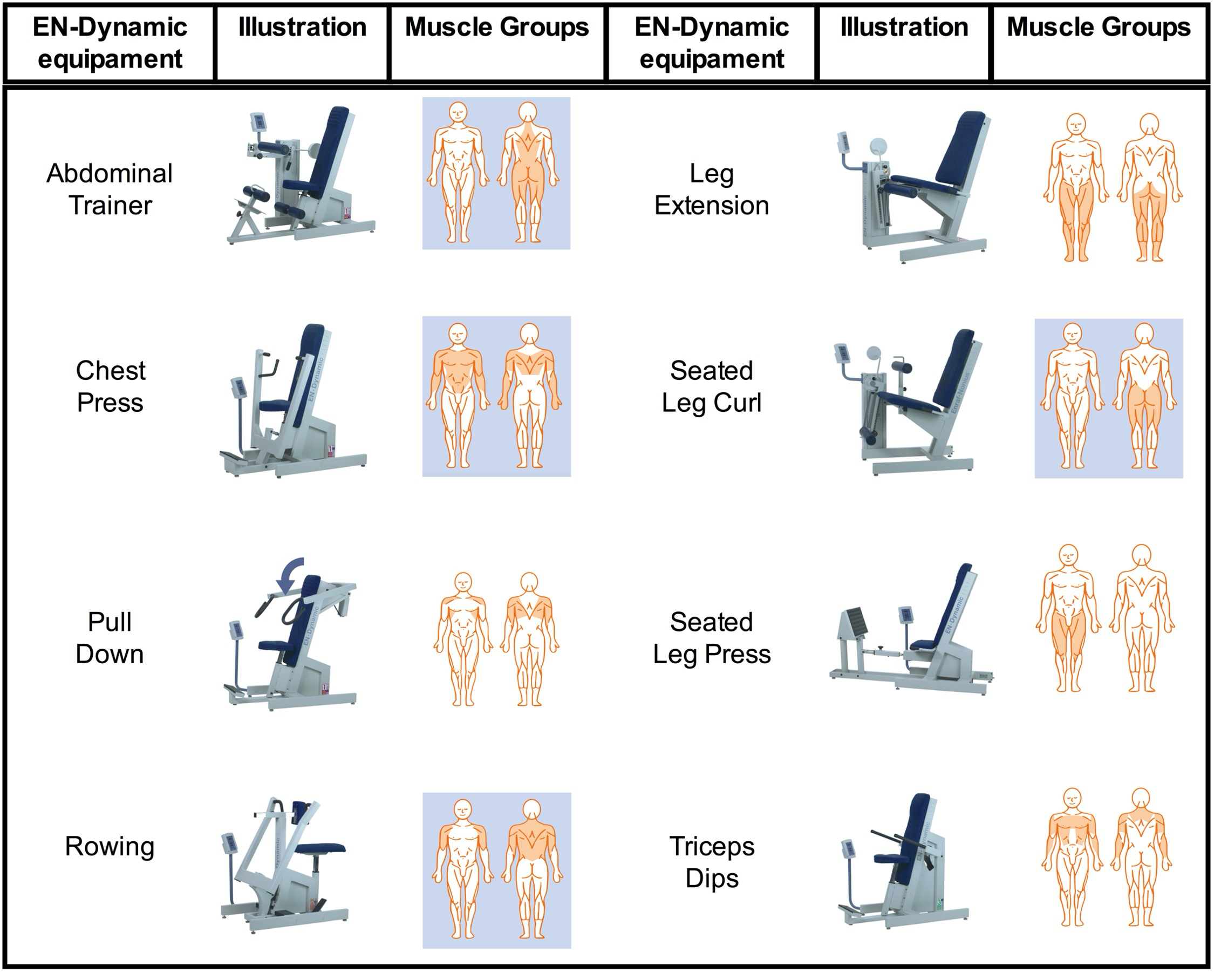
^

The HR was monitored continuously (Polar H7 sensor and Polar Beat app, Polar Electro Inc., Kempele, Finland) and (IPAD Air 2, Apple, Cupertino, USA) during CRT and the recovery period.^32^ The systolic blood pressure (SBP) and diastolic (DBP) were measured using an automated oscillometric calibrated device (OMROM MIT Elite plus, OMROM Health Care Inc, IL, USA) before and after each CRT session.

***Description of the techniques of MRT***

The MRT was based on the treatment described in the study of Jardine WM et al.[14] The evaluations and treatments were performed by an osteopath with five years of experience. The evaluation was performed initially with tests and palpations in specific areas of the body to evaluate the mobility and collect basic information on tissue characteristics (also known as TART– for Tenderness, Asymmetry, Range of motion change, Tissue texture change) to identify patterns of stiffness/tenderness of the tissues (muscles, fascias, etc). The evaluation also included tests such as sitting diaphragm evaluation, sphenobasilar symphysis (SBS) 'listening', sacral 'listening', pelvic floor evaluation, and global femoral artery evaluation. The evaluation was performed bilaterally, but treatment techniques were performed only on the side where restriction was found.

The MRT consisted of six selected osteopathy techniques (cranial, myofascial, and visceral techniques). Each technique was performed for 2 minutes with patients mostly supine, with an entire completed session lasting 15 minutes, 1x/week for 12 weeks.

Voluntaries in UHBR participated in the unsupervised home-based rehabilitation program that consisted of five reunions in three months. The subjects received guides about aerobic and resistance exercises to be performed at home, orientation about core components topics, including 1) exercise and heart rate monitoring, 2) nutrition, 3) pharmacology, 4) biopsychosocial behavior, and 5) risk factors. The patients were asked about their home exercise at each reunion, and any doubt was remedied. After 12 weeks of the CRT program and the home-based program, all subjects were submitted to the same test.

| Table 1. Description of the Myofascial Release Techniques | |
| --- | --- |
| 1.Thoracic diaphragm release technique | Bilateral hand contact in the subcostal region was applied to move the thorax into flexion/extension and lateral rotation to identify the area of greatest resistance. The direction identified to have the greatest resistance underwent a stretch and held until release of the tension after which the thorax was returned to the initial position with the patient sitting. |
| 2.Tentorium cerebelli release technique | A reciprocal tension maintaining a bilateral external rotation of the temporal bones was performed with the dura mater moved in cephalic traction and held until release with the patient in the supine position with a pillow under the knees. |
| 3. Pelvic ﬂoor release technique | Palpation of the sacrum and low back was performed while focusing on breathing. During each expiration one sacrum traction was performed to the side with the greatest resistance and repeated until fascial release. The patient was supine with knees flexed. |
| 4.Iliac fascial release technique | The release of the iliac fascia was performed with the fingers of the therapist’s hands pressing on the anterior superior iliac spine in a diagonal, inferior, and medial direction while the patient was supine and knees in flexion. The pressure was held until release. |
| 5. Femoral artery release technique | After the therapist localized the femoral artery both hands were placed around it and provided both a cranial and caudal pressure after which the direction identified to have the greatest resistance underwent a stretch and was held until release. The patient was supine with a pillow under the knees. |
| 6.Balancing the three diaphragms technique | One hand in the thoracic region (subcostal) and the other on the temporal bone allowed each breath to be palpated after which synchronization of diaphragms was performed using gentle movement of both hands to facilitate optimal breathing. The procedure was repeated with one hand in the chest (mid-sternal) and the other near the pelvic floor on the sacrum. This was repeated in each position until synchronization of the subcostal, mid-sternal, and pelvic floor was achieved. The patient was in the supine position with a pillow under the knees |
